# Cyclophilin A promotes HIV-1 reverse transcription but its effect on transduction correlates best with its effect on nuclear entry of viral cDNA

**DOI:** 10.1186/1742-4690-11-11

**Published:** 2014-01-30

**Authors:** Alberto De Iaco, Jeremy Luban

**Affiliations:** 1 Department of Microbiology and Molecular Medicine, University of Geneva, Geneva 1211, Switzerland; 2 Program in Molecular Medicine, University of Massachusetts Medical School, 373 Plantation Street, Biotech II, Suite 319, Worcester, MA 01605, USA

**Keywords:** HIV-1, Cyclophilin A, Cyclosporine A, Capsid, Nuclear transport, Virion uncoating

## Abstract

**Background:**

The human peptidyl-prolyl isomerase Cyclophilin A (CypA) binds HIV-1 capsid (CA) and influences early steps in the HIV-1 replication cycle. The mechanism by which CypA regulates HIV-1 transduction efficiency is unknown. Disruption of CypA binding to CA, either by genetic means or by the competitive inhibitor cyclosporine A (CsA), reduces the efficiency of HIV-1 transduction in some cells but not in others. Transduction of certain cell types increases significantly when CypA binding to particular HIV-1 CA mutants, i.e., A92E, is prevented. Previous studies have suggested that this cell type-specific effect is due to a dominant-acting, CypA-dependent restriction factor.

**Results:**

Here we investigated the mechanism by which CypA regulates HIV-1 transduction efficiency using 27 different human cell lines, 32 HeLa subclones, and several previously characterized HIV-1 CA mutants. Disruption of CypA binding to wild-type CA, or to any of the mutant CAs, caused a decrease in HIV-1 reverse transcription in all the cell lines analyzed here. This block to reverse transcription, though, did not correlate with cell type-specific effects on transduction efficiency. The level of 2-LTR circles, a marker for nuclear transport of the viral cDNA that results from reverse transcription, correlated closely with effects on infectivity. No correlation was observed between the cell type-specific effects on infectivity and the steady-state CypA protein levels in these cells. Instead, as indicated by a fate-of-capsid assay, CsA released the HIV-1 CA core from an apparent state of hyperstabilization, in a cell type-specific manner.

**Conclusion:**

These data demonstrate that, while CypA promotes reverse transcription under all conditions tested here, its effect on HIV-1 infectivity correlates more closely with effects on nuclear entry of the viral cDNA. The data also support the hypothesis that a cell-type specific CypA-dependent restriction factor blocks HIV-1 replication by delaying CA core uncoating and hindering nuclear entry.

## Background

The peptidyl-prolyl isomerase cyclophilin A (CypA) modulates human immunodeficiency virus type 1 (HIV-1) replication. The HIV-1 Gag precursor polyprotein (Pr55^gag^) associates with CypA during virion assembly and incorporates the host isomerase into nascent viral particles [1-5]. The CypA binding site maps to the capsid (CA) domain of Pr55^gag^, in particular to CA residues glycine 89 and proline 90 located in an exposed, proline-rich loop [1,3,6,7]. CypA also interacts with mature HIV-1 cores [3,7]. Though CypA catalyzes the isomerization of CA proline 90 *in vitro*, it is not clear if this isomerase activity of CypA is required for the modulation of HIV-1 replication, or if simple interaction with CA is sufficient [8-10].

The relevance of the CA-CypA interaction for HIV-1 replication has been demonstrated using multiple experimental approaches, including disruption of binding with the competitive inhibitor cyclosporine A (CsA), CA mutants G89V and P90A, CypA mutants in the active site, and depletion of endogenous CypA by gene knock-out or RNA knock-down [11-14]. The conclusions from experiments using each of these methods are in agreement with each other.

CypA binding to HIV-1 CA promotes an early step in the HIV-1 replication cycle in specific target cells, including MT4, CEM, 293 T, HOS, TE671, Jurkat T cells, and primary human CD4^+^ T cells [11,15-20]. HIV-1 replication in other target cells, including HeLa and H9 T cells, is independent of CypA [12,15]. Serial passage of HIV-1 in CD4^+^-HeLa cells, in the presence of a competitive inhibitor of the CA-CypA interaction, selected for HIV-1 CA mutants A92E and G94D [21]. HIV-1 viruses bearing either the A92E or G94D CA mutation are defective for replication in HeLa or H9 T cells, but disruption of the CypA-CA interaction rescued infectivity of these mutants in these cells [12,15,16,19]. Interestingly, CA mutations N74D and A105T have no effect on the CA-CypA interaction, but, when encoded in *cis*, either mutant will rescue the infectivity defect of the A92E mutant [22-26]. In addition, N74D and A105T render WT HIV-1 dependent on CypA-CA binding in H9 and HeLa cells, host cells that do not otherwise require CypA for WT HIV-1 replication [24,27]. When infecting 293 T, HOS, TE671, or Jurkat T cells, A92E and G94D render HIV-1 replication independent of CypA [12,16,17,19,28].

How CypA promotes HIV-1 infection or contributes to the block to A92E or G94D mutant virus replication in certain cell lines is not well understood. More specifically, it is not clear at which step of HIV-1 replication CypA inhibits A92E and G94D mutant viruses. Some groups have shown that the replication block is before completion of reverse transcription [16,29], but others have shown that it acts during later steps [17,30]. Some groups propose that CypA modulates HIV-1 CA core disassembly and the level of CypA expression in the target cell determines the proper timing of the process [15,17,19,29,31]. However, others were unable to confirm this [16,18]. Some experiments suggest that CypA is involved in the selection of the nuclear entry pathway of the virus [32]. Finally, heterokaryon experiments revealed a dominant, CypA-dependent restriction activity in HeLa cells [16]. Here we revisit the role of the CypA-CA interaction on HIV-1 replication, exploiting a large panel of different cell lines, select CA mutants, and an improved assay for HIV-1 nuclear entry that distinguishes 2-LTR circles from autointegrants [26].

## Results

### Cell-type specific effects of CsA on HIV-1 transduction correlate more closely with CypA KD than do CA mutants that block CypA binding

The CypA-CA interaction can be disrupted with the competitive inhibitor CsA or by mutation of CA amino acids important for binding [1,3]. Before assessing a large panel of cell lines for the cell type-specific effects of CypA on HIV-1 transduction the effect of CsA or of CypA-binding loop mutants was compared to the effect of CypA KD in HeLa and Jurkat, two cell lines previously shown to have very different CypA phenotypes [12,15].

HeLa and Jurkat cells were transduced with lentiviral KD vectors that encode miRNAs engineered to knockdown either CypA mRNA or firefly luciferase control, as previously described [8,12,23,33]. The primary transcript containing the miRNA also encodes puromycin N-acetyltransferase, so transduced cells were selected in pools in the presence of puromycin. The two stable KD cell lines were challenged with VSV G-pseudotyped, three-part HIV-1-GFP reporter vectors bearing either wild-type CA or the A92E CA mutation. These and all virus stocks used here were normalized by reverse transcriptase activity.

**Figure 1:**
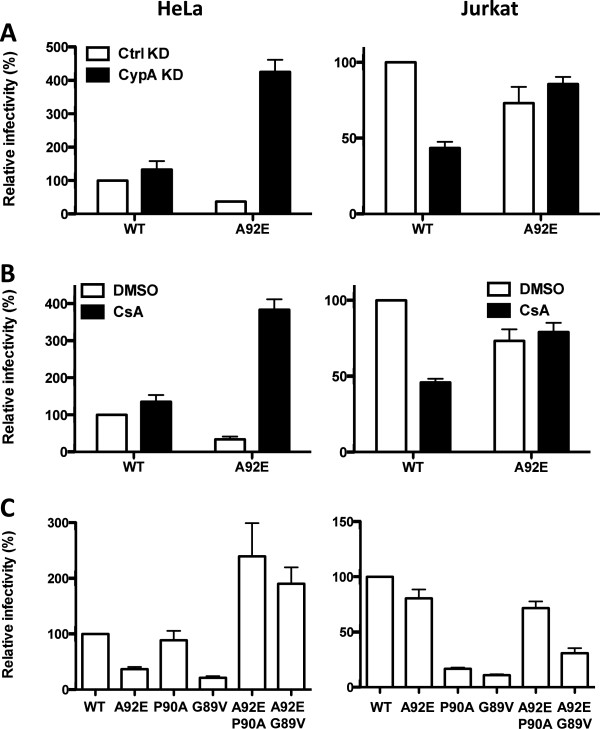
**Effects of CsA on HIV-1 transduction correlate more closely with CypA KD than do CA mutants that block CypA binding.** HeLa cells (left) or Jurkat cells (right) were challenged with HIV-1 GFP reporter viruses bearing wild-type (WT) or mutant CA. 72 hours later GFP expression was assessed by flow cytometry. The cells were **(A)** depleted of endogenous CypA or **(B)** pre-treated with 5 μM CsA or DMSO and challenged with WT or A92E CA mutant virus. **(C)** Cells were challenged with viruses bearing the indicated capsid mutations (P90A, G89V, P90A/A92E, G89V/A92E). Data represent one of at least three independent experiments. Error bars represent ± SEM (n = 3).

72 hrs after transduction GFP expression was assessed by flow cytometry as a measure of transduction efficiency (Figure 1A). As a control that GFP reporter activity resulted from infection and not from passive transfer of GFP protein, target cells were also challenged with an HIV-1 vector bearing two RT mutations (D185K and D186K) that render the viral polymerase catalytically inactive (data not shown). As previously reported, CypA KD inhibited WT CA transduction in Jurkat about 2-fold, but there was no inhibition of WT vector in HeLa cells [8,12,17]. In contrast, the A92E CA mutant virus was 3-fold lower than WT CA in the control HeLa cells, while depletion of CypA increased its transduction around 10-fold. In Jurkat T cells, CypA KD had no effect on A92E virus replication.

The effect of 5 μM CsA on WT and A92E CA mutant transduction of HeLa and Jurkat T cells was tested next (Figure 1B). In each condition, when the target cells were incubated in CsA the effect was identical to that observed with CypA KD. The effect of CA mutants G89V and P90A was tested next (Figure 1C). G89V inhibited transduction around 10-fold in both cell lines and, when combined with A92E, transduction was rescued in HeLa cells but not Jurkat. P90A inhibited transduction of Jurkat T cells, but there was no effect in HeLa. Combination with A92E rescued P90A transduction in both cell lines. This analysis clearly shows that P90A and G89V CA mutations have pleiotropic effects, inhibiting HIV-1 transduction to a greater extent than simple disruption of the CypA/CA interaction. In light of these results, CsA was used in the subsequent experiments examining the CA/CypA interaction in a panel of cell lines.

### CypA interaction with HIV-1 CA universally promotes reverse transcription

**Figure 2:**
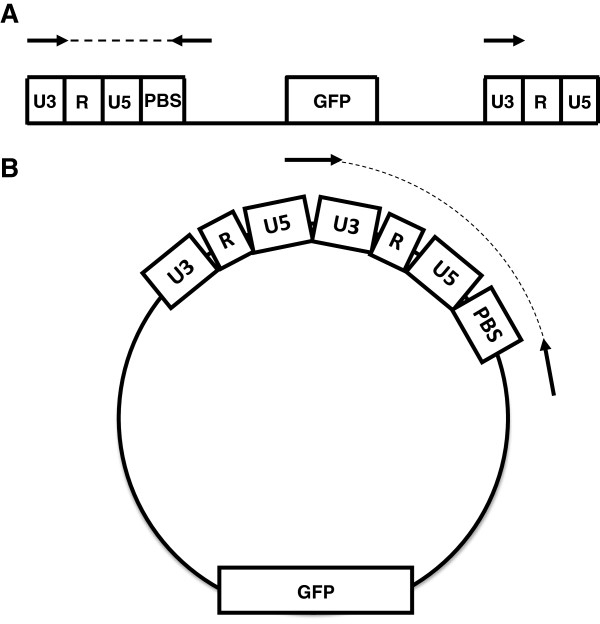
**Schematic for strategy to amplify viral cDNA and 2-LTR circles. (A)** Schematic for the amplification of the final product of reverse transcription. This PCR identifies HIV-1 cDNA formed after the “second jump” of the reverse transcription process. **(B)** Schematic for the amplification of 2-LTR circles. A forward primer spanning the junction LTR-LTR was used in order to discriminate 2-LTR circles from products of autointegration. The arrows indicate the primers used in the reaction, and the dashed lines indicate the final PCR products.

Using PCR primers that detect full-length viral cDNA (Figure 2A) several groups have reported that CypA promotes HIV-1 infectivity at a stage in the replication cycle before the completion of reverse transcription [11,16,17,29]. In contrast, disagreement exists among different labs regarding which step in the viral replication cycle is inhibited by CypA interaction with the A92E or G94D mutant CAs [16,17,29]. Recently, to clarify the role of TNPO3 in HIV-1 nuclear entry, we developed a PCR assay that is specific for the 2-LTR circles that form when the viral cDNA enters the nucleus [34]. Unlike PCR primers that are commonly used to detect 2-LTRs [35], these primers hybridize directly to the LTR-LTR junction (Figure 1B), and therefore distinguish *bona fide* 2-LTR circles from autointegrants [23,26].

**Figure 3:**
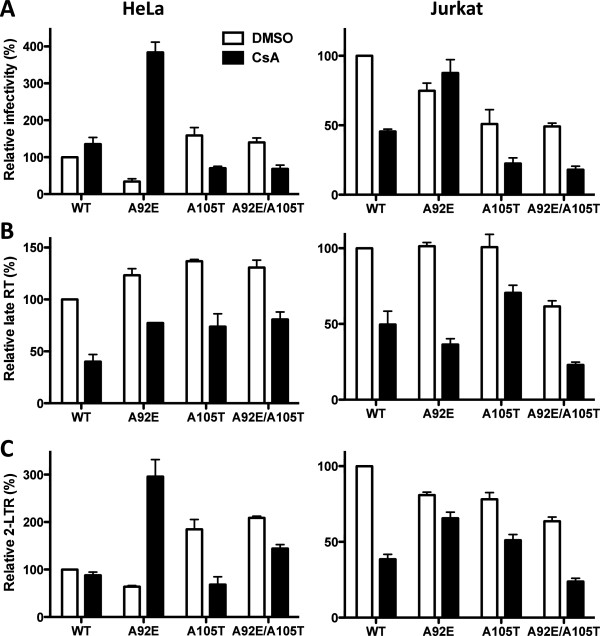
**CypA promotes HIV-1 reverse transcription in both HeLa and Jurkat T cells, but inhibits nuclear entry in HeLa cells.** HIV-1 reporter viruses bearing wild-type (WT) CA or the indicated CA mutants, were used to challenge HeLa (left) or Jurkat T (right) cells, treated with 5 μM CsA or DMSO as control. 72 hours later GFP expression was assessed by flow cytometry **(A)**. 24 hrs later, late reverse transcription **(B)** and 2-LTR circles **(C)** were assayed by quantitative PCR. Data represent one of at least three independent experiments. Error bars represent ± SEM (n = 3).

To understand the role of CypA in HIV-1 replication, we examined the effect of CsA on HIV-1 infectivity, reverse transcription, and nuclear entry (Figure 3). HeLa and Jurkat T cells were treated with 5 μM CsA or DMSO as control, and challenged with WT, A92E, A105T or A92E/A105T CA mutant viruses (Figure 3). A105T and A92E/A105T CA mutants were included in the analysis because they confer sensitivity to CsA in H9 cells [27]. D185K/D186K RT double mutant virus was used as a control to demonstrate that signal in the PCR reactions required *de novo* viral cDNA synthesis in the target cells and did not result from contaminating DNA. In each case, the PCR products with the RT mutant were at least 100-fold lower than for the wild-type virus (data not shown).

As a measure of infectivity, GFP expression from the reporter gene was assessed 72 hrs after infection (Figure 3A). CsA had no effect on WT HIV-1 transduction of HeLa cells but inhibited transduction of Jurkat T cells 2-fold. The infectivity of HIV-1 bearing the CA A92E mutation was increased 11-fold in HeLa cells. In Jurkat T cells, the A92E mutant conferred resistance to the inhibitory effect of CsA. The CA A105T mutant virus replicated like the WT in HeLa cells and was inhibited 2-fold in the presence of CsA. On Jurkat T cells, the A105T mutant was less infectious than the WT, but the magnitude inhibition by CsA was similar to that of the WT. When combined *in cis*, the A105T mutant rescued infectivity of the A92E mutant virus in HeLa cells, but conferred sensitivity to CsA. CA mutant A105T therefore seems to have a dominant effect over the A92E mutation.

The effect of CsA on the efficiency of reverse transcription (Figure 2A) by WT, A92E, A105T, and A92E/A105T CA mutant viruses was assessed acutely after infection of HeLa and Jurkat T cells (Figure 3B). Interestingly, CsA caused a modest reverse transcription defect of similar magnitude for all the viruses, in both HeLa and Jurkat T cells. Inhibition of HIV-1 reverse transcription by CsA correlated with effects on infectivity of WT virus in Jurkat T cells and for A105T and A92E/A105T mutant viruses infecting either HeLa or Jurkat (compare Figure 3A and B). In contrast, the inhibition of reverse transcription by CsA did not correlate with the infectivity effects for WT CA in HeLa cells or for A92E infecting either HeLa or Jurkat T cells. In all cases then, CA interaction with CypA increased the level of reverse transcription.

### The CypA-CA interaction inhibits HIV-1 nuclear entry in a cell specific manner

Given the discrepancy between the effects of CsA on infectivity and reverse transcription, the formation of 2-LTR circles was assessed by quantitative PCR (qPCR) 24 hrs after transduction (Figure 3C). A primer annealing to the 2-LTR junction was used (Figure 2B); PCR reactions with this primer amplify only *bona fide* 2-LTR circles, not the products of autointegration [26].

Relative to the amount of viral cDNA formed by either WT or A92E, CsA increased the levels of 2-LTR circles (Figure 3B and C). CsA increased the efficiency of nuclear entry, compensating for the 2-fold block in reverse transcription of WT virus, and making the A92E CA mutant 5-fold more infectious than it was under control conditions in HeLa cells. The A105T CA mutant rendered WT and A92E viruses insensitive to the effect of CsA on the nuclear entry of the viral cDNA. Though the A105T mutation prevents CypA-mediated nuclear entry inhibition without blocking the CypA/CA interaction [23], this CA mutant still requires CypA for optimal reverse transcription. Additionally, CsA increased A92E viral 2-LTR circles in Jurkat cells to a lesser extent than it did in HeLa cells, indicating that CypA binding to CA inhibits HIV-1 nuclear entry to different extents in HeLa and Jurkat T cells.

### Influence of the CypA-CA interaction on HIV-1 infectivity in a panel of cell lines

**Figure 4:**
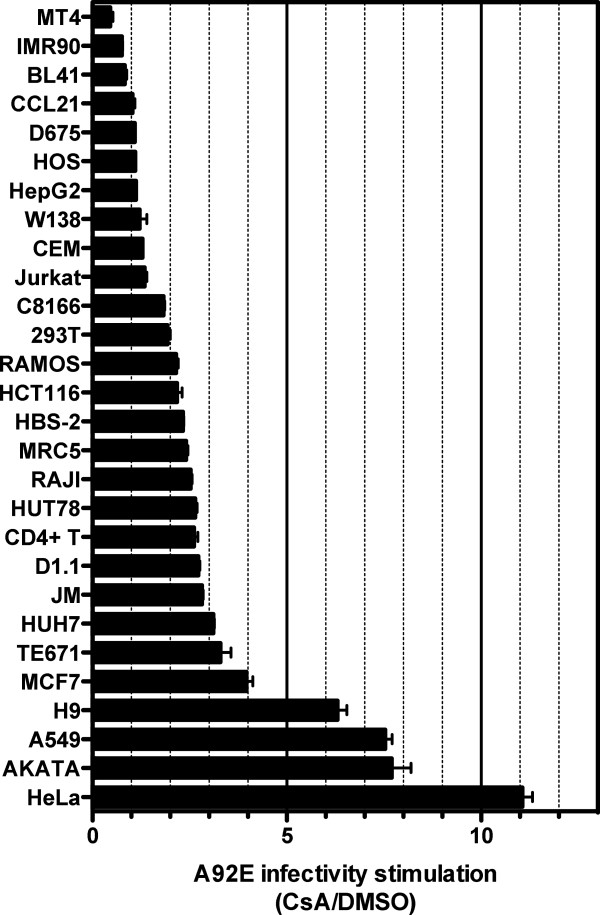
**Variable effect of CsA on transduction of different cell lines by HIV-1 bearing the A92E CA mutant.** A panel of 27 cell lines and human CD4+ T cells were treated with 5 μM CsA or DMSO as control and challenged with an HIV-1-GFP reporter vector bearing the A92E CA mutation. 72 hrs post-transduction the percent GFP^+^ cells was determined by flow cytometry, as an indicator for infectivity. The ratio of A92E mutant virus infectivity in CsA vs DMSO treated cells is shown. Data represent one of at least two independent experiments. Error bars represent ± SEM.

The CypA-CA interaction inhibited nuclear entry of the A92E CA mutant to different extents in HeLa and Jurkat T cells. To further examine the effect of CsA on A92E transduction, 27 cell lines and primary CD4^+^ T cells were treated with 5 μM CsA, or DMSO as control, and challenged with the three-part HIV-1 vector bearing the A92E mutation (Figure 4). 72 hrs after transduction, expression of the GFP reporter was assessed by flow cytometry. As previously reported [16,17,19,21,29], A92E infection of HeLa and H9 cells was increased significantly when the CypA-CA interaction was disrupted (11-fold and 6-fold, respectively, Figure 4). A92E replication was increased 7-fold by CsA in the human Burkitt’s lymphoma derived B-cell line AKATA [36] and in the human alveolar adenocarcinoma epithelial cell line A549 [37]. Enhancement of A92E replication by CsA was between 2 to 4-fold in 13 cell lines tested and in the primary CD4^+^ T cells. Transduction of 9 cell lines was not significantly altered by CsA. Finally, CsA inhibited A92E virus transduction of the human T cell line MT4 [38] by about 2-fold. The effect of CsA on A92E transduction therefore varied over a large range in the 27 cell lines tested (Figure 4).

### Influence of the CypA-CA interaction on HIV-1 nuclear entry, as assessed in a panel of cell lines

**Figure 5:**
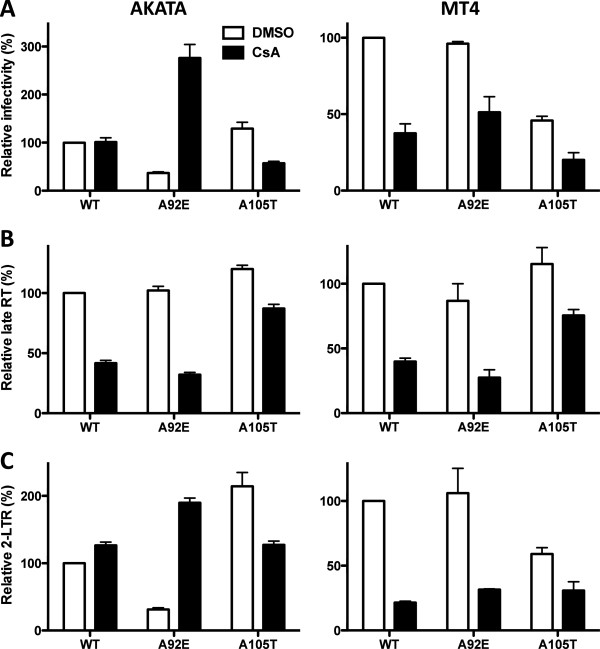
**CypA promotes HIV-1 reverse transcription in both AKATA and MT4 cells, but inhibits nuclear entry in AKATA cells.** HIV-1 reporter viruses bearing wild-type (WT) CA or the indicated CA mutants, were used to challenge AKATA (left) or MT4 (right) cells, treated with 5 μM CsA or DMSO as control. 72 hours later GFP expression was assessed by flow cytometry **(A)**. 24 hrs later, late reverse transcription **(B)** and 2-LTR circles **(C)** were assayed by quantitative PCR. Data represent one of at least three independent experiments. Error bars represent ± SEM (n = 3).

Two of the 27 cell lines tested above, AKATA and MT4 cells, were selected to assess the effect of CsA on the synthesis and nuclear entry of HIV-1 cDNA acutely after infection (Figure 5). AKATA and MT4 cells were transduced with WT, A92E, or A105T viruses. GFP expression was tested 72 hrs later as a marker of viral replication (Figure 5A). The effect of CsA on transduction of AKATA cells was similar to that in HeLa cells: WT virus was insensitive to CsA, A92E infectivity was enhanced, while infectivity of A105T was inhibited. In contrast, transduction of MT4 cells by WT, A92E, or A105T was inhibited by CsA.

The formation of late reverse transcription products and 2-LTR circles was assessed next in AKATA and MT4 cells treated with CsA. Reverse transcription of WT, A92E, or A105T viruses, in the setting of acute infection of either AKATA or MT4 cells was inhibited by CsA treatment (Figure 5B). In AKATA cells treated with CsA, WT virus infection was rescued at the level of nuclear entry (Figure 5C). Nuclear entry of the A92E virus was inhibited compared to WT virus and the CsA treatment rescued the block. Nuclear entry of none of the tested viruses was altered significantly compared to cDNA levels when MT4 cells were used for the analysis.

**Figure 6:**
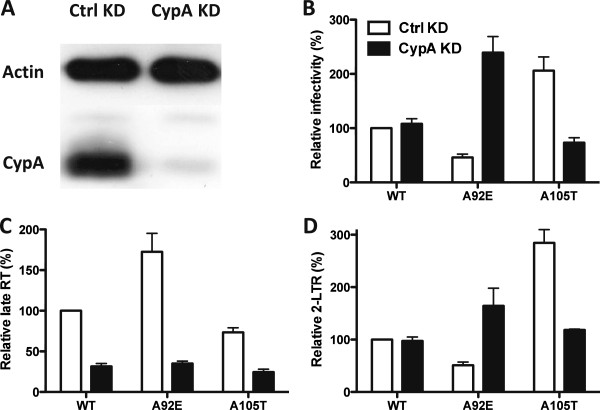
**The effect of CypA KD in AKATA cells on HIV-1 transduction. (A)** CypA level in AKATA cells transduced with a lentiviral vector expressing an shRNA targeting CypA, or control (Ctrl). Cell lysates were probed in western blot with antibodies against β-actin (upper panel) or CypA (lower panel). CypA and Ctrl KD AKATA cells were challenged with WT or CA mutant HIV-1 reporter viruses. 72 hours later GFP expression was assessed by flow cytometry **(B)**. 24 hrs after infection, late reverse transcription **(C),** or 2-LTR circles **(D),** were assayed by quantitative PCR. Data represent one of at least three independent experiments. Error bars represent ± SEM (n = 3).

To confirm that the effect of CsA on A92E nuclear entry resulted from CypA blockade, endogenous CypA protein was depleted from AKATA to nearly undetectable levels using the lentiviral miRNA vectors (Figure 6A). A92E transduction (Figure 6B) and nuclear entry (Figure 6D) were enhanced by CypA KD in AKATA cells, reproducing the phenotype seen with transduction in the presence of CsA (Figure 5).

### Influence of the CypA-CA interaction on HIV-1 nuclear import, as assessed in a panel of HeLa cell clones

**Figure 7:**
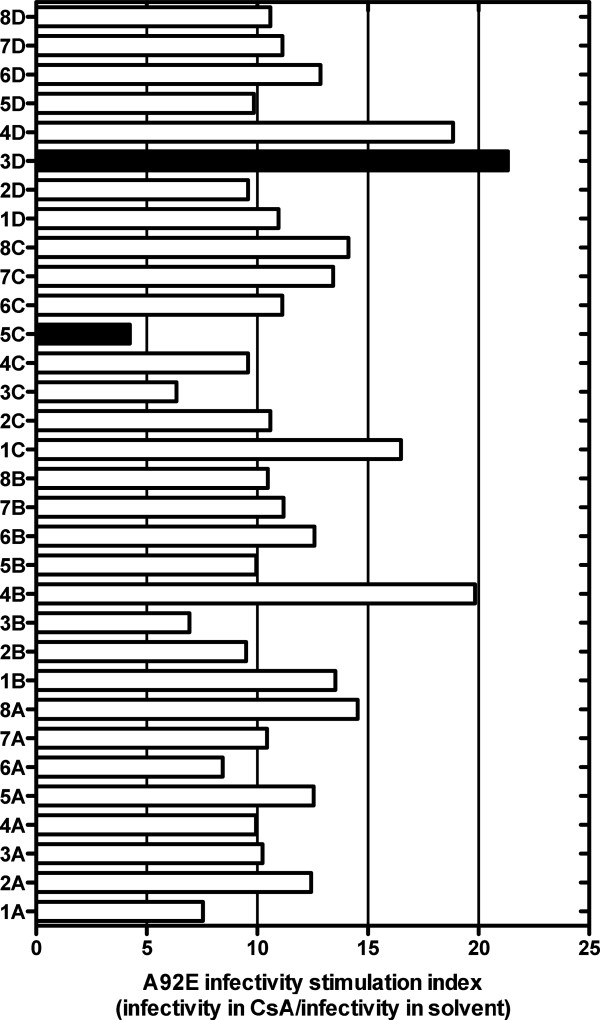
**Variable effect of CsA on transduction of different HeLa cell clones by HIV-1 bearing the A92E CA mutation.** 32 HeLa cell clones were treated with 5 μM CsA or DMSO as control and challenged with an HIV-1-GFP reporter vector bearing the A92E CA mutation. 72 hrs post-transduction the percent GFP^+^ cells was determined by flow cytometry, as an indicator for infectivity. The ratio of A92E mutant virus infectivity in CsA vs DMSO treated cells is shown. Data represent one of at least two independent experiments. Error bars represent ± SEM.

A92E vector was more dependent on CsA in HeLa cells than in any of the other 27 cell lines tested (Figure 4). HeLa cell transduction by A92E was ~3-fold lower than by the WT, and CsA enhanced its infectivity around 11-fold (Figure 3A). The variable permissivity of target cells cloned by limiting dilution [39,40] was exploited next to obtain 32 HeLa cell clones in which the stimulation of A92E transduction by CsA ranged from 4 to 22-fold (Figure 7).

**Figure 8:**
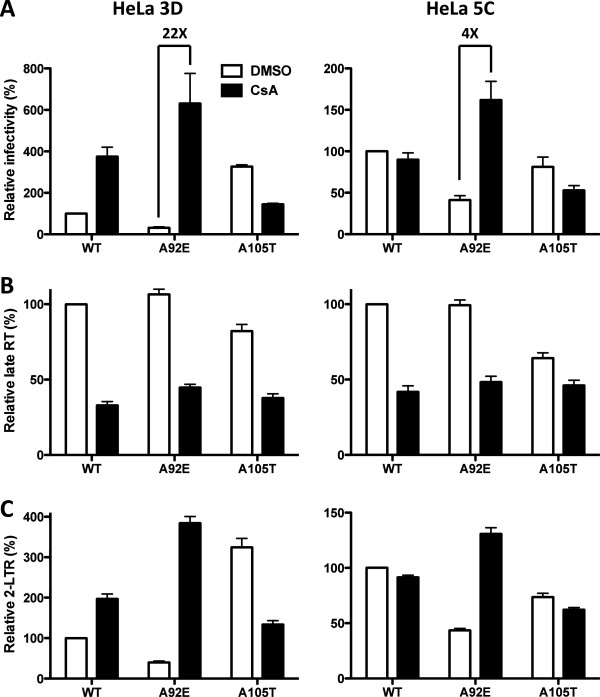
**The effect of CsA on transduction of HeLa cell clones 3D and 5C by HIV-1.** WT or CA mutant HIV-1 reporter viruses were used to challenge HeLa 3D (left) or 5C (right) clones treated with 5 μM CsA or DMSO as control. 72 hours later GFP expression was assessed by flow cytometry **(A)**. 24 hours later, late reverse transcription **(B)** and 2-LTR circles **(C)** were assayed by quantitative PCR. Data represent one of at least three independent experiments. Error bars represent ± SEM (n = 3).

The two clones with the most extreme effects of CsA on A92E transduction, 3D and 5C, were selected for further testing. In the HeLa 3D clone, CsA enhanced A92E transduction 22-fold and WT transduction 4-fold (Figure 8A). In the HeLa 5C clone, A92E transduction increased only 4-fold in the presence of CsA; WT virus infectivity was not altered. A105T transduction was inhibited in the 3D clone as in the parental HeLa cell line but in the 5C clone the magnitude of inhibition by CsA was reduced. HIV-1 vectors transducing HeLa 3D and 5C clones showed stable phenotypes up to 3 months after clone derivation (data not shown).

The efficiency of reverse transcription (Figure 8B) and of 2-LTR circle formation (Figure 8C), after challenge of the 3D and 5C HeLa clones with WT, A92E, and A105T vectors, was examined next. CsA blocked the accumulation of late reverse transcripts by all three vectors in both HeLa cell clones (Figure 8B). In all cases, CsA increased the level of 2-LTR circles to the same extent that it increased transduction efficiency (Figure 8C). In the HeLa 3D clone, even 2-LTR accumulation by the WT vector was increased in the presence of CsA. These results indicate that CsA promotes transduction efficiency by promoting nuclear entry of the HIV-1 pre-integration complex.

### Modulation of HIV-1 nuclear entry is not determined by CypA protein level

Some previous studies indicate that the cells in which CsA enhances A92E replication contain higher levels of CypA compared to cells in which CsA has no effect on A92E replication [15-19,29]. Other studies, offer evidence that a dominant, CypA-dependent restriction factor is expressed in the cell lines in which replication of the A92E is CsA-dependent [16,19].

**Figure 9:**
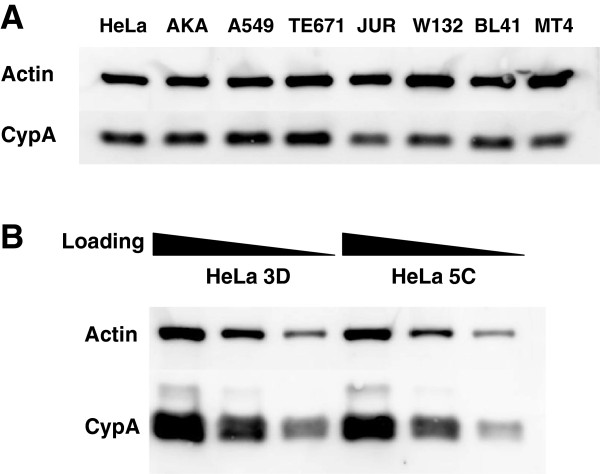
**CypA protein level in different cell lines does not correlate with CsA-dependency.** CypA protein levels by western blot in HeLa, AKATA (AKA), A549, TE671, Jurkat T (JUR), W132, BL41 and MT4 cell lines **(A),** and in HeLa 3D and 5C clones **(B)**. Loading was normalized by cell count. In B, samples were serially diluted 4-fold. Cell lysates were probed in western blots with anti-CypA antibody (upper panel) and anti-β-actin antibody (lower panel).

To determine whether increased amounts of CypA protein correlates with the effects of CsA on A92E infectivity, CypA protein levels were analyzed by western blot in 3 cell lines where A92E transduction is highly stimulated by CsA (HeLa, AKATA, and A549), 3 cell lines where A92E transduction is moderately enhanced (TE671, Jurkat T and W132 cells), and 2 cell lines where A92E is inhibited by CsA (BL41 and MT4). Loading of the gel was normalized by number of cells and by western blot for actin. CypA protein levels varied slightly but there was no correlation with the CsA phenotype (Figure 9A). Similarly, no consistent difference in CypA protein levels was observed when the 3D and 5C HeLa clones were compared to each other (Figure 9B).

### CypA increases HIV-1 CA core stability

Previous work has shown a role for CypA in the uncoating of HIV-1 CA cores [29,31]. Li et al. used an *in vivo* assay to demonstrate that CypA either stabilizes or destabilizes HIV-1 cores, depending on the specific target cell [29]. Shah et al. showed that CypA inhibits HIV-1 CA core disassembly in vitro [31].

**Figure 10:**
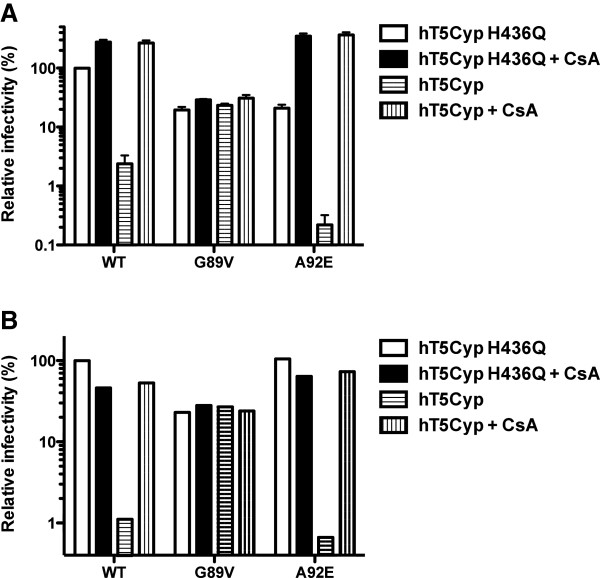
**Effect of hT5Cyp on HIV-1 transduction.** HeLa clone 3D **(A)** and MT4 cells **(B),** stably expressing the WT or mutated (H436Q) hT5Cyp restriction factor, were treated with CsA or DMSO as control, and challenged with HIV-1-GFP reporter viruses bearing WT, G89V or A92E mutant capsid. Expression of GFP was checked by flow cytometry 72 hrs after challenge with the virus. Infectivity relative to WT virus is represented. Data represent one of at least three independent experiments. Error bars represent ± SEM (n = 3).

To assess the *in vivo* stability of WT and A92E CA cores after acute infection in the presence of CsA, HeLa clone 3D and MT4 cells were used as target cells. As controls for conditions under which CA cores have preciously characterized stabilities, each of these cell lines was transduced to express either human TRIM5 fused to human CypA (hT5Cyp) or a non-restrictive mutant that has the CA binding site in CypA disrupted (hT5Cyp-H436Q) [41]. hT5Cyp is a restriction factor modeled after the *Aotus* TRIMCyp gene [39]. hT5Cyp potently restricts HIV-1 reverse transcription, most likely by destabilizing the HIV-1 CA core [42]. As expected, WT and A92E viruses were restricted by hT5Cyp in HeLa clone 3D (Figure 10A) and in MT4 (Figure 10B). In the case of both cell lines, disruption of the CypA catalytic site in hT5Cyp (H436Q), mutation of an HIV-1 CA amino acid important for interaction with CypA (G89V), or the competitive inhibitor CsA, prevented restriction activity (Figure 10).

**Figure 11:**
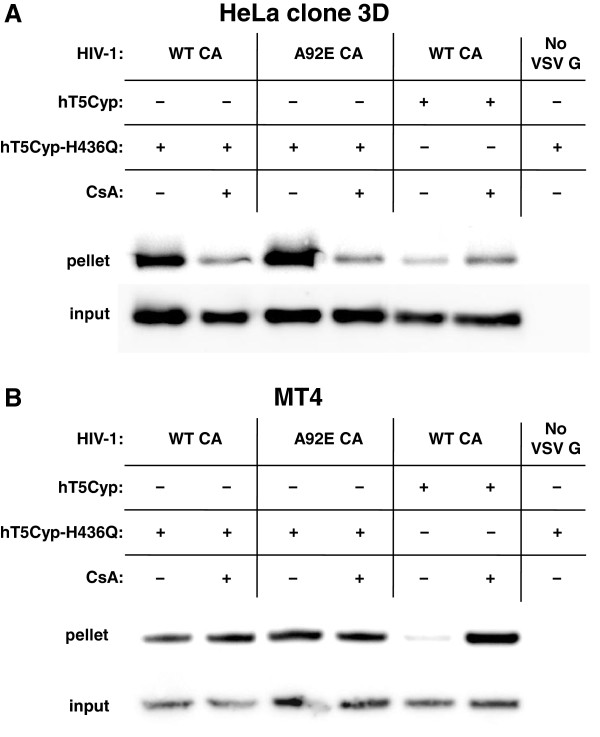
**The CypA-CA interaction hyper-stabilizes HIV-1 CA cores in a target cell-specific fashion.***env*-minus HIV-1, pseudotyped with VSV G, and bearing either WT or A92E mutant CA, was incubated with HeLa clone 3D **(A)** or MT4 cells **(B)** stably expressing hT5Cyp or hT5Cyp-H436Q, as indicated. Cells were treated with CsA or DMSO as control, for 16 hrs. As a control, to demonstrate, that signals in this assay required viral entry, virions lacking VSV G were used. The target cells were lysed and the cytoplasmic fraction (input) was pelleted through a 50% sucrose cushion to obtain the particulate fraction (pellet). The fractions were then analyzed by western blot with anti-p24 antibody. All experiments are representative of at least 2 repetitions.

HeLa clone 3D bearing hT5Cyp-H436Q was challenged with envelope-minus HIV-1 pseudotyped with VSV G, bearing either WT or A92E CA, in the presence of CsA or DMSO as solvent control. 16 hrs post-transduction, cells were lysed and the cytoplasmic extract was accelerated through a 50% sucrose cushion. As compared with WT CA, the recovery of A92E CA in the pellet was significantly increased (Figure 11A). CsA decreased the amount of CA protein recovered in the pellet for both WT and A92E CA. hT5Cyp also decreased the CA yield in the pellet, an effect that was reversed by CsA to the level seen in the absence of any CypA/CA interaction. Virus without VSV G was used as a control for CA that had been taken up by cells non-specifically. These results indicate that the CA-CypA interaction inhibits HIV-1 nuclear entry by stabilizing the virion core.

To determine if increased CA core stability also explains the increased reverse transcription due to CypA, similar CA recovery experiments were conducted with MT4 cells (Figure 11B); CsA decreased reverse transcription in these cells without increasing nuclear entry. In MT4 cells bearing hT5Cyp-H436Q the recovery of A92E CA in the pellet was similar to that of the WT CA, and CsA caused no change for either. CA recovery was greatly decreased in MT4 cells bearing hT5Cyp, and this effect was blocked by CsA. Additionally, identical results were observed in the parental cells that did not express hT5Cyp (data not shown). Thus, while effects of CypA on nuclear entry correlated with effects of CypA on CA core stability (Figure 11A), the effects of CypA on reverse transcription did not correlate in any detectable way with effects on core stability (Figure 11B).

### CsA blocks reverse transcription at 6 hrs post infection

**Figure 12:**
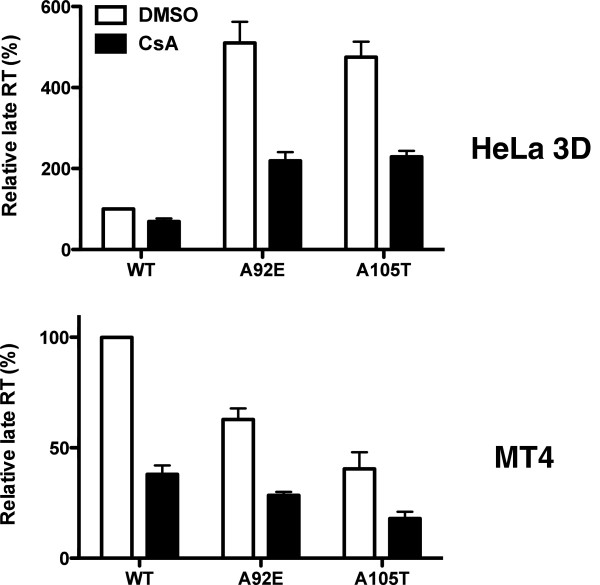
**CsA inhibits reverse transcription 6 hrs after transduction.** HeLa 3D clone and MT4 cells were challenged with HIV-1 CA mutant reporter viruses. 24 hours later, late reverse transcription was assayed by quantitative PCR. Data represent one of at least three independent experiments. Error bars represent ± SEM (n = 3).

The peak accumulation of HIV-1 full-length linear cDNA occurs between 6 and 12 hrs post-infection [35]. Given that HIV-1 cDNA was evaluated 24 hrs after infection in the above experiments, the effect of CsA was assessed at an earlier time point to see if conclusions were the same. HeLa 3D and MT4 cells were transduced with WT, A92E, and A105T capsid mutants in the presence or absence of CsA. 6 hrs later, the level of late reverse transcription products was assessed (Figure 12). In each case, CsA treatment of the target cell resulted in a reduction of full-length linear HIV-1 cDNA.

## Discussion

### Host cell CypA promotes HIV-1 reverse transcription

Previous studies showed that CypA is required for HIV-1 replication in specific cell types, but not in others [11,15-19]. In addition, it was shown that the requirement of CypA for HIV-1 was after virus entry but prior to reverse transcription [11,16,17,29]. The experiments reported here demonstrate that interaction between CypA and CA is important for the completion of HIV-1 reverse transcription in cells where the CA-CypA interaction has no detectable effect on infectivity, or even inhibits it.

How CypA promotes reverse transcription remains unclear. Li et al. showed that CypA regulates optimal stability of the HIV-1 CA core in Jurkat T cells and that disruption of the CypA-CA interaction results in premature uncoating [29]. Since the process of CA core uncoating is associated with reverse transcription, such CA destabilization would be expected to result in the inhibition of virus replication [43]. However, the experiments here showed that HIV-1 transduction of Jurkat T cells was partially defective for nuclear entry in the presence of CypA. When HIV-1 was tested on MT4 cells, the only cell line where CypA favors HIV-1 reverse transcription without effect on nuclear entry, CsA had no significant effect on the WT CA stability. However, the assay for in vivo CA stability might not be sensitive enough to detect the small block in reverse transcription caused by CsA. Using an *in vitro* assay, Shah et al. detected a stabilizing effect of CypA on HIV-1 CA [31].

### CypA inhibits HIV-1 nuclear entry by promoting CA core stability

HIV-1 carrying either A92E or G94D CA mutations were selected by serial passage of HIV-1 on CD4^+^-HeLa cells in the presence of CsA [21]. It remains unclear at which step of the HIV-1 replication cycle CsA enhances replication of these mutant viruses [16,17,29,30]. Using a recently optimized PCR assay to quantify HIV-1 2-LTR circles [23,26], it was determined here that CsA stimulates replication of the A92E CA mutant, and to a lesser extent the WT virus, at a step between the formation of the viral cDNA and its transport into the nucleus. It was also demonstrated that the block to nuclear entry correlates with increased yield of particulate HIV-1 CA from the cytoplasm of acutely infected cells. These results suggest that when CypA interacts with HIV-1 CA it delays virion uncoating. It was recently shown that another cellular factor, CPSF6, can bind to HIV-1 CA, stabilize CA cores, and block nuclear entry of HIV-1 cDNA, in a similar fashion [26].

### A92E sensitivity to CsA is highly variable in different cells

Replication of A92E virus was previously described to be dependent on CsA in HeLa and H9 cells, and independent from CsA in MT4, CEM, 293 T, HOS, TE671 and Jurkat T cells [11,15-19]. Here the replication of A92E in the presence of CsA was assessed in 8 cell lines that had previously been characterized and in 19 additional cell lines. Results here show that the effect of CsA on the mutant virus was highly variable. MT4 was the only cell line analyzed in which A92E was sensitive to the block in reverse transcription in presence of CsA, but totally independent of the drug at a subsequent step in the replication cycle. In contrast, A92E reverse transcription was decreased when the CypA-CA interaction was disrupted in 293 T, HOS, CEM, TE671 and Jurkat T cells, but this block was rescued at the level of nuclear entry. It is also interesting to note how different A92E behaves in the presence of CsA when replicating in very similar cell lines such as the Burkitt lymphoma B-cell lines AKATA and BL41, or in the T-cell lines H9 and MT4.

To understand if CypA protein levels explain the different sensitivity of A92E to CsA in different cell lines the CypA protein levels were measured in 8 cell lines where A92E behaved differently in presence of CsA (Figure 9). Some slight differences in CypA expression were detectable, but they did not correlate with the CsA-dependence in those cell lines.

These results reinforce the hypothesis that a CypA-dependent restriction factor is differentially expressed in different cell lines [16,19]. Song et al. previously analyzed heterokaryons formed by fusing CsA-dependent HeLa cells and CsA-independent 293 T cells, and showed that the CsA-dependence of the A92E and G94D mutants was dominant, suggesting the presence of a cellular restriction factor [16]. The results here indicate that WT HIV-1 is restricted at the level of nuclear entry in some cell lines by an unknown restriction factor that also requires CypA. Moreover, the A92E CA mutation renders HIV-1 more sensitive to this putative restriction factor.

### Are CPSF6 or MX2 the CypA-dependent restriction factors?

Disruption of CypA-CA interaction with competitive inhibitors or mutation of viral CA restores the infectivity of WT and A92E mutant viruses [22]. The A105T mutation renders viruses with WT or A92E CA independent of the restriction on nuclear entry without preventing binding of CypA to CA [23]. A105 might prevent recognition by the putative restriction factor, independently of the CypA-CA interaction. Recently, CA mutant A105T was described for its ability to prevent restriction of HIV-1 replication by a truncated form of the human protein CPSF6 [26]. Like the unknown CypA-dependent restriction factor proposed here, CPSF6 hyperstabilizes the HIV-1 CA core, blocking transduction at a step between reverse transcription and nuclear entry. These effects have only been observed when CPSF6 is forced to accumulate in the cytoplasm and have never been reported with the endogenous protein under physiologic conditions. Nonetheless, CPSF6 might be responsible for the inhibition of HIV-1 replication prior to nuclear entry in a CypA-dependent manner. Supporting this idea, Shah et al. showed that CsA reduces the inhibitory effect after depletion of TNPO3 [31]. Recent studies described a new HIV-1 restriction factor, myxovirus resistance 2 (MX2) [44-46]. MX2 is an interferon-induced protein that blocks nuclear entry of HIV-1 in a CA-dependent manner [44]. CypA depletion or mutation on CA preventing CypA binding (G89V) prevent HIV-1 restriction mediated by MX2 [46]. Further analysis of the role of CPSF6 and MX2 in CsA-treated cells is required to determine the role these host factors in the phenotypes reported here.

## Conclusions

Since the first report in 1993 of direct binding between HIV-1 CA and CypA, many studies have tried to characterize the functional significance of this interaction. CypA was shown to influence HIV-1 reverse transcription and later steps of viral replication in a cell type-dependent manner, but the results were often different in different labs. Here the mechanism of action of CypA was revisited with improved qPCR techniques to assess the efficiency of reverse transcription and nuclear entry of a panel of CA mutants transducing 27 different cell lines and 32 HeLa clones in the presence of the CypA inhibitor CsA. CypA promoted HIV-1 reverse transcription under all conditions, but inhibited HIV-1 nuclear entry in a cell type-dependent manner. Moreover, evidence is provided that CypA facilitates HIV-1 restriction by a dominant-acting restriction factor that stabilizes CA cores.

## Methods

### Cell lines, tissue culture, primary cells, and drugs

HeLa, 293 T, TE671, W132, IMR90, CCL21, HOS, HepG2, MRC5, MCF7 and A549 cells were grown in Dulbecco’s modified Eagle medium (DMEM) (Invitrogen) supplemented with 10% fetal bovine serum (FBS) (PAA), 20 mM L-glutamine, 1000 U/ml penicillin, and 1000 mg/ml Streptomycin (GIBCO). MT4, Jurkat T, BL41, HUT78, AKATA, HBS-2, JM, H9, D1.1, C8166, RAJI, RAMOS, D675 and CEM were grown in Roswell Park Memorial Institute medium (RPMI) (Invitrogen) supplemented with 10% fetal bovine serum (FBS) (PAA), 20 mM L-glutamine, 1000 U/ml penicillin, and 1000 mg/ml Streptomycin (GIBCO). Human CD4^+^ T cells were purified by EasySep CD4+ T Cell Enrichment Kit (StemCell Technologies) and cultured in X-Vivo 15 medium (Lonza) in the presence of IL-2 (30 U/ml). Transduction with lentiviral vectors was performed 3 days after stimulation with Dynabeads human T-activator CD3/CD28 (Lifetechnologies). Cyclosporine A (CsA, Sigma-Aldrich) was used at a concentration of 5 μM.

### Plasmids

pWPTs-GFP is an HIV-1-based transfer vector with EGFP expression under the control of the EF1α promoter [47]. p8.9NdSB is a minimal HIV-1 packaging plasmid for *gag* and *pol* expression. p8.9NdSB bearing G89V, G89V/A92E, P90A, P90A/A92E, A92E, A92E/A105T and A105T in the CA sequence were previously described [23]. pMD2-G encodes the vesicular stomatitis virus G protein (VSV-G) [48]. pAPM is an HIV-1 based knockdown vector in which a single transcript driven by the spleen focus-forming virus (SFFV) LTR contains a miR30 framework modified to target a gene of interest and the puromycin N-acetyltransferase gene [33]. pAPM-CypA and pAPM-control were used to knock-down respectively CypA or control mRNA [49]. hT5Cyp and hT5Cyp-H436Q viral vectors were previously described [41].

### Production of viruses and vectors

Viruses and minimal vectors were produced by transfection of 293 T cells using Polyethylenimine (PEI) (Sigma, Inc), and normalized for reverse transcriptase activity (SGPERT) [23].

### Reverse transcriptase assay (SGPERT)

Reverse transcriptase activity in the supernatants was quantified using a Sybr green I-based real-time PCR enhanced reverse transcriptase assay (SGPERT) that possesses both high sensitivity and an extraordinary dynamic range [23]. The assay is a modified version of one described previously [50]. Briefly, virions in cell-free supernatant were disrupted by adding an equal volume of SGPERT lysis buffer containing 0.25% Triton X-100, 50 mM KCl, 100 mM TrisHCl pH7.4, 0.4 U/μl RNase inhibitor (RiboLock, MBI Fermentas). Lysed virions were used for reverse transcription of MS2 RNA template (Roche) [51]. Quantification of reverse transcribed products was carried out in a CFX96 thermal cycler (Biorad) using Sybr-Green I, hotstart Taq and reaction buffer (Fermentas), and an MS2 primer set as already described [51]. A standard curve was obtained using known concentrations (expressed in functional units) of recombinant HIV-1 RT (Ambion).

### Generation of stable KD cell lines

HeLa, Jurkat T and AKATA cells were transduced with pAPM microRNA-based shRNA vectors targeting either control or CypA mRNA. Cells were selected with 2 to 10 μg/mL of puromycin two days after transduction.

### Western blot analysis

For western blot analysis we used rabbit anti-CypA antibody (Enzo Life Sciences), human anti-p24 (NIBSC) and mouse anti-actin antibody (Sigma). The secondary antibodies were HRP-linked donkey anti-rabbit IgG (GE Healthcare Life Sciences), HRP-linked donkey anti-human IgG (Jackson) and HRP-linked sheep anti-mouse IgG (GE Healthcare Life Sciences).

### Quantitative PCR for late reverse transcriptase products and 2-LTR circles

Low molecular weight DNA was extracted from 4 × 10^6^ cells using the QIAprep Spin Miniprep Kit (Qiagen), following the manufacturer’s instructions. Late RT analysis was previously described [23].

The primers used for the 2-LTR circle detection are: Junct2 fwd, 5′- CAGTGTGGAAAATCTCTAGCAGTAC-3′ [44], coupled with pWPT J2 rev, 5′-GCCGTGCGCGCTTCAGCAAGC-3′ [23]. 2-LTR circles qPCRs with TaqMan probe detection method were made using the primers (Butler 2001): MH535, 5′-AACTAGGGAACCCACTGCTTAAG-3′; and MH536. 2-LTR circle PCR reaction mix contained 1× Sybr green mix (10 mM Tris pH 8.3, 10 mM KCl, 2.5 mM NH_4_SO_4_, 5 mM MgCl_2_, 0.1 mg/ml BSA, 0.2 mM dNTPs, 1x Sybr green), 300 nM each primer, 6 μl of template low-molecular weight DNA, and 0.2 μl of Hot Start Taq Polymerase (Promega) in a volume of 20 μl. After initial incubation at 95°C for 2 min to activate the Hot Start Taq Polymerase, 40 cycles of amplification and acquisition were carried out at 95°C for 6 s, followed by 10 s at 55°C, 30 s at 72°C and 6 s at 80°C.

Where indicated, cells were treated 1 hour before infection with 5 μM CsA.

### Fate of capsid assay

Fate of capsid assay was performed as previously described [44]. Hela clone 3D and MT4 cells expressing hT5Cyp or hT5Cyp-H436Q were seeded onto T75 flasks. 24 hours later, the confluent cells were incubated with 10 ml of Env- HIV-1, pseudotyped with VSV G, and bearing either WT or A92E mutant CA, in presence of 5 μM CsA for 30 min at 4°C and then shifted to 37°C. After 4 hours, the virus was removed, the cells were washed and returned to 37°C in presence of 5 μM CsA for 12 hours. Cells were detached with pronase (7 mg/ml in DMEM) for 5 min at 4°C, washed 3 times with ice cold PBS and finally resuspended in 2.5 ml of hypotonic lysis buffer (10 mM Tris-HCl, pH 8.0, 10 mM KCl, 1 mM EDTA). After 5 min incubation on ice, the cells were lysed in a 7-ml Dounce homogenizer by 15 stokes with pestle B. The lysate was cleared by centrifugation for 3 min at 3,000 rpm at 4°C to remove the nuclear fraction. 100 μl of the cleared lysate was collected to determine the viral input in the assay, while 2 ml was layered on top of a 7-ml 50% sucrose gradient and centrifuged for 2 hours at 30,000 rpm at 4°C using a Beckman SW41 rotor. After centrifugation, the pellet was resuspended in 1× SDS-PAGE loading buffer. Samples were analyzed by WB using antibody against p24.

### Single-cell cloning

HeLa cells were trypsinized and counted. Cells were seeded in 96 well plates such that there was one cell per every 5 wells. Growth of isolated clones was checked by bright field microscopy.

## Competing interests

The authors declare that they have no competing interests.

## Authors’ contributions

AD and JL conceived and designed the experiments and wrote the paper. AD performed the experiments. Both authors read and approved the final manuscript.
